# Severe Transfusion-Dependent Anemia in Hereditary Hemorrhagic Telangiectasia: A Critical Case of Gastrointestinal Bleeding and Pulmonary Arteriovenous Malformation

**DOI:** 10.7759/cureus.106476

**Published:** 2026-04-05

**Authors:** Mahika Shetty, Milind S Awale, Shravan Gangula, Jyothi R Patri

**Affiliations:** 1 Research, Drexel University College of Medicine, Philadelphia, USA; 2 Medical Management, Carnegie Mellon University, Pittsburgh, USA; 3 Hospital Medicine, West Virginia University, Wheeling Hospital, Wheeling, USA; 4 Family and Community Medicine, Coffeyville Regional Medical Center, Coffeyville, USA; 5 Lifestyle Medicine, Heritage Valley Family Medicine Residency Program, Beaver Falls, USA

**Keywords:** gastrointestinal telangiectasia and bleeding, hereditary hemorrhagic telangiectasia (hht), high-output cardiac failure, pulmonary arteriovenous malformation (pavm), transfusion-dependent iron deficiency anemia

## Abstract

Hereditary hemorrhagic telangiectasia (HHT) is a genetic disorder of the vasculature associated with mucocutaneous telangiectasia and visceral arteriovenous malformations (AVMs). These manifestations can frequently lead to chronic blood loss and iron deficiency anemia. In some cases, anemia can be severe and transfusion-dependent, reflecting an advanced disease phenotype with persistent vascular involvement and cumulative blood loss.

This report presents a case of a 58-year-old female with a known diagnosis of HHT who presented with fatigue, dizziness, and chronic melena, and was found to have a hemoglobin level of 4.7 g/dL. Imaging revealed persistent pulmonary AVM disease, which may increase cardiopulmonary demand, particularly in the setting of severe anemia, and may contribute to complications if left untreated. The patient required transfusion of five units of packed red blood cells and underwent endoscopic ablation of multiple telangiectasias in the stomach and duodenum. This case underscores the persistent nature of HHT-related gastrointestinal bleeding and the cardiopulmonary consequences of multi-system AVM burden. It emphasizes the necessity for multidisciplinary care and ongoing surveillance in patients with HHT to prevent symptom progression.

## Introduction

Hereditary hemorrhagic telangiectasia (HHT), also referred to as Osler-Weber-Rendu syndrome, is a rare autosomal disorder with a global prevalence of approximately one in 5,000 individuals [1-3]. This condition involves fragile blood vessels prone to rupture and the development of abnormal vascular connections, leading to mucocutaneous telangiectasia and arteriovenous malformations (AVMs) in organs such as the gastrointestinal tract, liver, lungs, and brain [1-3].

These vascular abnormalities arise from dysregulated angiogenesis driven by pathogenic variants, most commonly in ENG, ACVRL1, and SMAD4 genes. These genetic mutations disrupt transforming growth factor‑β (TGF‑β) signaling, which leads to the formation of fragile vascular structures that are susceptible to bleeding [4].

Individuals with HHT frequently experience persistent epistaxis and gastrointestinal bleeding, which may result in chronic iron deficiency anemia [1,2]. Gastrointestinal bleeding becomes more prevalent with increasing age in patients with HHT, particularly after the age of 40, and may, in some cases, require intravenous iron therapy or blood transfusions [2,4,5]. Diagnosis is based on the Curacao criteria, which integrate genetic and clinical features: recurrent epistaxis, muco-cutaneous telangiectasia, a first-degree relative with HHT, and multi-organ AVMs [6]. These criteria highlight the genetic etiology underlying vascular fragility and abnormal vessel formation [4]. According to the Second International HHT Guidelines, systematic screening for pulmonary and cerebral AVMs is strongly recommended due to risks of paradoxical embolism and high-output cardiac failure [6]. Pulmonary arteriovenous malformations (PAVMs) create right‑to‑left shunting, which may result in hypoxemia and contribute to compensatory high‑output cardiac states in chronically anemic patients. In HHT, untreated PAVMs are associated with increased risk of stroke and brain abscess due to paradoxical embolization [7].

This report presents a patient with HHT who developed severe anemia secondary to chronic gastrointestinal bleeding. The case is notable for the profound degree of anemia, extensive multi-organ AVM involvement, and ongoing transfusion requirements despite prior ablation and embolization procedures.

## Case presentation

A 58-year-old female arrived at the emergency room (ER) with two days of symptoms consisting of weakness, fatigue, and intermittent black stools, which she reported as a chronic issue. She denied having any fever, headaches, palpitations, chest pains, shortness of breath (SOB), hematuria, hematemesis, or hemoptysis. The most important part of her past medical history was her long-standing HHT with severe chronic anemia, with a history of transfusion dependence requiring weekly blood transfusions over the past several months. She had previously undergone endoscopic evaluation with identification and treatment of gastrointestinal telangiectasias and had been managed with iron supplementation, though her anemia remained refractory.

Her past medical history included a colonoscopy and esophagogastroduodenoscopy (EGD) one year prior for AVM in the stomach, duodenum, and colon. It also included a pulmonary AVM embolized in the past. Her father had a history of HHT and chronic anemia.

Upon arrival at the ER, her vital signs showed mild tachypnea, but she was alert and not in distress. Exams marked her as stable even though she looked pale. There was no active bleeding noted anywhere, and her abdominal and lung exams were normal. Her labs showed a severely low hemoglobin of 4.7 g/dL along with microcytic anemia and low iron saturation. Leukopenia and mild thrombocytopenia were also present, raising the possibility of additional contributing factors such as nutritional deficiency, chronic disease, or bone marrow suppression, although no acute etiology was identified during hospitalization. Table 1 summarizes vital signs and initial laboratory results on arrival to the hospital.

**Table 1:** Summary of vital signs, physical exam, and laboratory results on admission. This table summarizes the patient’s initial clinical presentation, including vital signs, physical examination findings, and laboratory values at the time of hospital admission, highlighting severe anemia with borderline microcytic indices and iron studies suggestive of iron deficiency in the setting of chronic gastrointestinal blood loss in the setting of HHT. Abbreviations: MCV = mean corpuscular volume; WBC = white blood cells; RBC = red blood cells; TSAT = transferrin saturation; TIBC = total iron-binding capacity; AST = aspartate aminotransferase; ALT = alanine aminotransferase; CBC = complete blood count; CMP = comprehensive metabolic panel; HHT = hereditary hemorrhagic telangiectasia. Source: Table synthesized by MS and JRP.

Type of test	Test	Findings	Reference range
Vitals	Heart rate	96 bpm	60-100 bpm
	Blood pressure	109/46	110-130/70-90
	Respiratory rate	24/min	12-20/min
	Temperature	98°F	97-99°F
Physical exams	Appearance	Pale	N/A
	Lungs	Clear	Clear
	Abdomen	Soft, non-tender	N/A
	Skin	No active bleeding	N/A
CBC	Hemoglobin	4.7 g/dL	12-16 g/dL
	Hematocrit	14.4%	36-46%
	Platelets	130,000 mm^3^	150000-400,000 mm^3^
	WBC count	1.4/mm^3^	4,500-11,000/mm^3^
	MCV	79.9 µm^3^	80-100 ​​µm^3^
	RBC count	1.8 million/mm^3^	3.5-5.5 million/mm^3^
Iron studies	Iron level	34 mcg/dL	28-182 mcg/dL
	TSAT	11%	11-46%
	Ferritin	25.9 ng/mL	11-307 ng/mL
	TIBC	317	280-400 mcg/dL
CMP	Potassium	3.7 mEq/L	3.7-5.2 mEq/L
	Creatinine	0.5 mg/dL	0.8-1.4 mg/dL
	Albumin	2.6 g/dL	3.9-5.0 g/dL
	AST	21 IU/L	10-34 IU/L
	ALT	13 IU/L	8-37 IU/L

**Figure 1:**
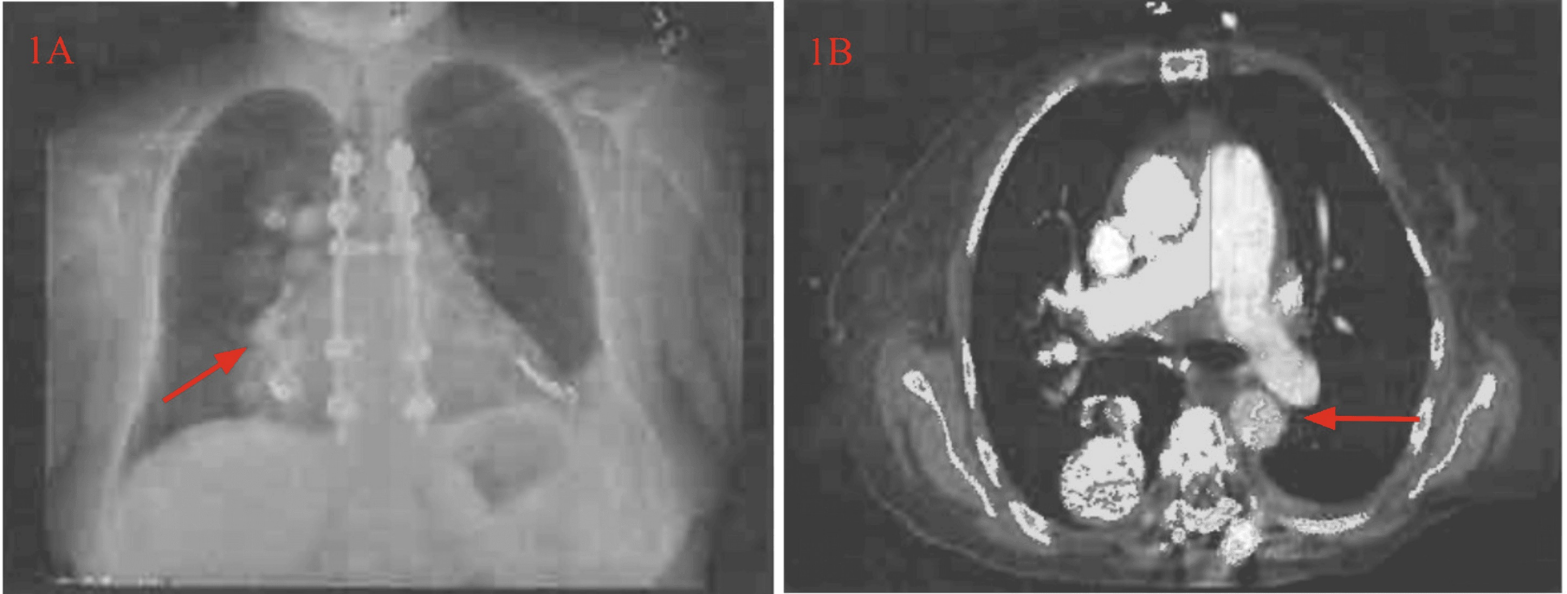
Chest X-ray and CT angiography demonstrating a right pulmonary arteriovenous malformation. Figure A shows a chest X-ray with a right perihilar opacity consistent with a pulmonary arteriovenous malformation (AVM). Figure B shows a CT angiogram confirming a large right-sided pulmonary AVM, which may contribute to increased cardiopulmonary demand in the setting of severe anemia. Source: Figure synthesized by MS and JRP with images extracted from the patient's scans.

Imaging studies included a chest X-ray and chest computed tomography angiography (CTA). Figure 1A presents a chest X-ray demonstrating a large right-sided density attributable to the patient’s known pulmonary AVMs. Figure 1B depicts a CTA confirming a large, incompletely embolized pulmonary AVM on the right.

**Figure 2:**
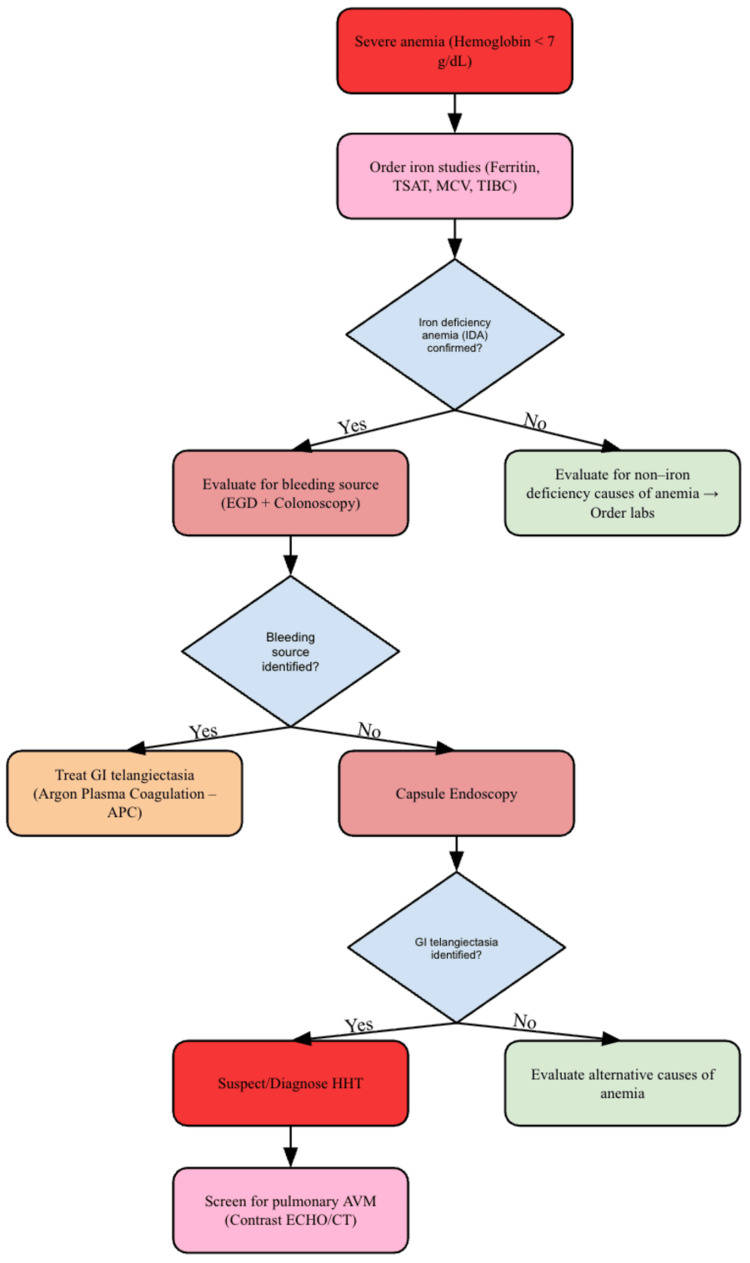
Diagnosis of severe anemia in HHT. This flowchart illustrates a general approach to anemia evaluation in HHT; however, in patients with established HHT, management should prioritize bleeding control, iron repletion, and consideration of advanced therapies for refractory disease. This framework reflects general clinical practice and was applied in the evaluation of the presented patient. Abbreviations: IDA = iron deficiency anemia; TSAT = transferrin saturation; MCV = mean corpuscular volume; TIBC = total iron-binding capacity; GI = gastrointestinal; EGD = esophagogastroduodenoscopy; APC = argon plasma coagulation; HHT = hereditary hemorrhagic telangiectasia; AVM = arteriovenous malformation; ECHO = echocardiogram; CT = computed tomography. Source: Flowchart created by MS and JRP with data extracted from [3].

The patient was admitted, received five units of packed red blood cells (RBCs), and was started on intravenous proton pump inhibitor (PPI) therapy. A multidisciplinary approach was implemented for treatment. Gastroenterology performed EGD and treated multiple telangiectasias distributed throughout the stomach and proximal duodenum using argon plasma coagulation (APC), without identification of a single actively bleeding lesion. Given the high prevalence of small bowel involvement in HHT, further evaluation with capsule endoscopy was considered in the setting of ongoing or recurrent bleeding. Interventional radiology was consulted to assess candidacy for repeat embolization. On hospital day three, the patient was discharged in a stable condition with prescriptions for ferrous sulfate, PPI, and vitamin B12. There were plans for continued multidisciplinary management of HHT-related bleeding and consideration of escalation therapies if transfusion dependence persisted. Figure 2 displays the guideline-based treatment used for this patient.

## Discussion

This case is clinically significant because of the severity of the patient’s anemia and the combined effects of gastrointestinal (GI) bleeding and PAVM-related cardiac strain. A hemoglobin level below 5 g/dL is uncommon, even in patients with HHT, and suggests severe iron deficiency in the setting of chronic blood loss. Chronic bleeding from mucocutaneous and GI telangiectasias is one of the most frequent complications in HHT, and over time can result in transfusion-dependent anemia. Transfusion dependence in HHT reflects cumulative mucosal vascular burden and is associated with reduced quality of life and increased cardiopulmonary strain [8,9]. Although chronic blood loss is the primary driver of anemia in HHT, the presence of leukopenia and thrombocytopenia suggests that additional contributing factors may be present and should be considered.

HHT is an autosomal dominant vascular disorder characterized by progressive vascular dysplasia affecting multiple organ systems [1-3]. Clinical diagnosis relies on the Curacao criteria, which require the presence of at least three of the following symptoms: spontaneous recurrent epistaxis, mucocutaneous telangiectasias, visceral AVMs, or a first-degree relative with HHT [6]. In this case, the patient met diagnostic criteria based on her history of chronic bleeding, mucosal telangiectasias on EGD, visceral pulmonary AVMs, and a positive family history.

HHT results from mutations in genes involved in vascular development, which affect signaling pathways that regulate blood vessel formation. Since HHT follows an autosomal dominant inheritance pattern, first-degree relatives have a 50% risk of carrying the mutation [10]. Due to this, genetic counseling and screening of family members are important components of long-term management.

Chronic gastrointestinal bleeding is one of the most common complications in patients with HHT, particularly those over 40 years of age, and typically arises from small telangiectasias in the stomach or small intestine [5,8,9]. Research shows that gastrointestinal telangiectasias are present in a significant proportion of patients with HHT, particularly in older individuals and those with symptomatic disease, with some studies reporting prevalence rates approaching 80% [8,9]. HHT is secondary to defects in specific genes that help blood vessels grow and function as intended. When these genes are affected, dysregulation of the transforming growth factor-beta signaling pathway leads to impaired endothelial function, abnormal angiogenesis, and the formation of fragile vascular structures prone to rupture, as well as the development of direct arteriovenous connections [4]. Over time, this can lead to low iron stores and transfusion-dependent anemia [4].

**Figure 3:**
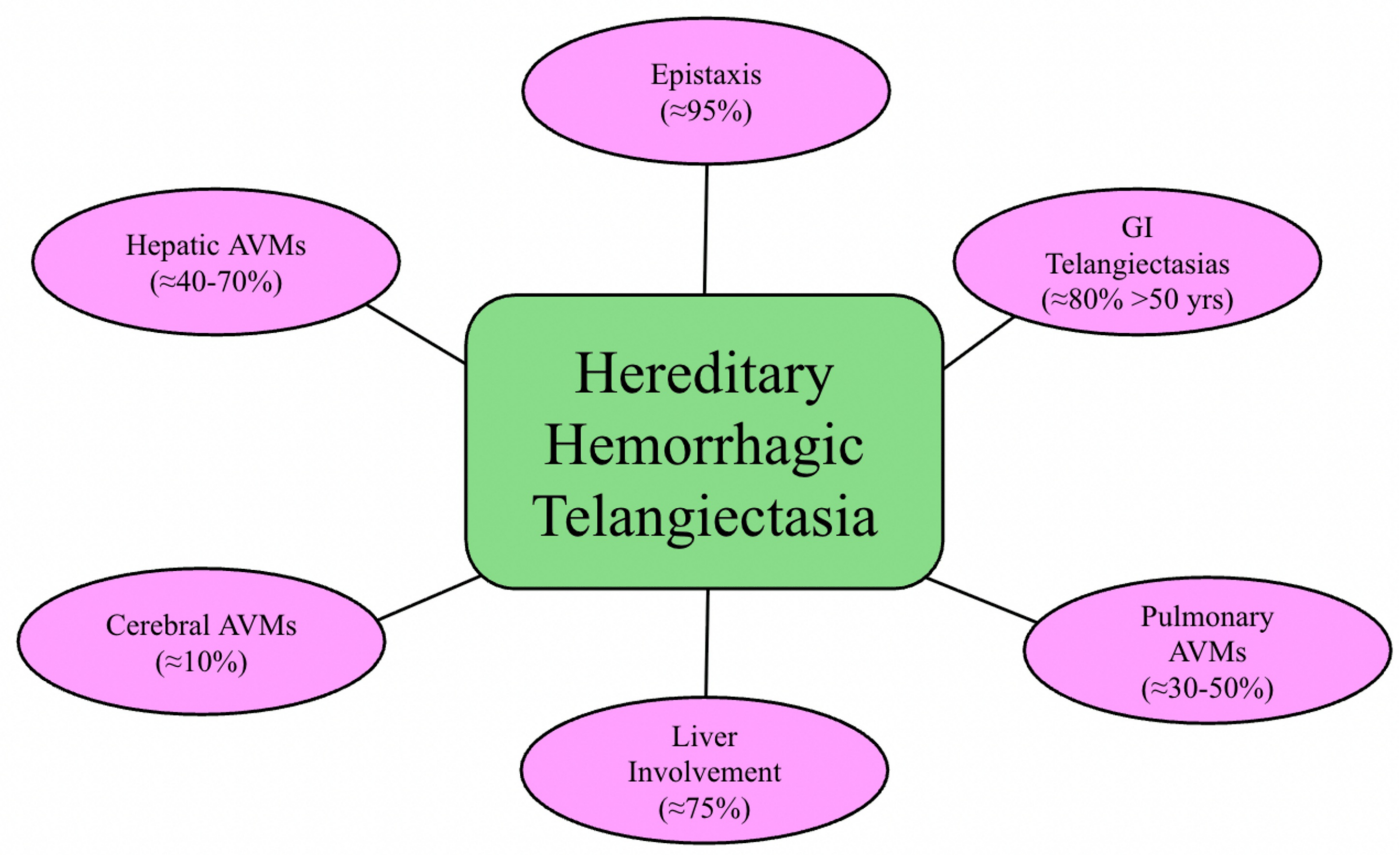
Main symptoms and organ involvement in hereditary hemorrhagic telangiectasia. This schematic summarizes common organ involvement and clinical manifestations in HHT, along with approximate prevalence rates reported in the literature. This figure represents general disease patterns and is not specific to the presented patient. Abbreviations: AVM = arteriovenous malformations; GI = gastrointestinal; HHT = hereditary hemorrhagic telangiectasia. Source: Figure created by MS and JRP with data extracted from [10].

Another major feature of HHT, displayed in Figure 3, is pulmonary AVMs, which are present in 35% of patients and can cause issues such as low oxygen levels and paradoxical emboli. They contribute to right-to-left shunting and may contribute to or exacerbate high-output cardiac physiology in patients with severe anemia [9,10]. In this case, the patient had a large right-sided pulmonary AVM that was only partially embolized, which is likely the reason for her rapid heartbeat. This, in addition to low hemoglobin, likely contributed to increased cardiac demand, raising concern for high-output physiology in the setting of severe anemia.

Management of HHT-related bleeding involves a multi-disciplinary approach. Standard treatment can include oral or IV iron replacement as well as blood transfusions when necessary. APC can decrease bleeding temporarily, but the lesions often recur, so repeated treatments are usually needed [11]. In patients with transfusion‑dependent anemia despite endoscopic therapy, systemic anti‑angiogenic therapy has demonstrated a reduction in epistaxis severity and transfusion requirements [12,13]. In more severe cases, systemic anti-angiogenic therapies such as thalidomide have proven to inhibit bleeding frequency by targeting abnormal vessel formations [14]. Emerging evidence also supports the use of anti-vascular endothelial growth factor (anti-VEGF) therapies, such as bevacizumab, which may reduce transfusion requirements and improve quality of life in patients with severe HHT [15]. Since HHT affects multiple organ systems, management typically requires a multidisciplinary approach involving gastroenterology, pulmonology, cardiology, interventional radiology, and genetic counseling services. Given the patient’s transfusion-dependent course despite prior endoscopic therapy, escalation to systemic therapy may be considered.

HHT does not undergo malignant transformation; the disease is very progressive, with increasing numbers of telangiectasias and AVMs developing over time. This can lead to worsening anemia, cardiopulmonary stress, and reduced quality of life. Patients often need repeated endoscopies, iron treatments, or even blood transfusions to keep their anemia under control because this chronic disease reappears in patients 46% of the time [16]. Published cohort studies have identified older age, multiple gastrointestinal telangiectasias, and recurrent need for endoscopic therapy as predictors of transfusion dependence in HHT, consistent with the clinical profile of the patient described in this report [8,16]. Early identification, such as the steps seen in Figure 3, is extremely important for patients with HHT to ensure that individuals do not develop all of these symptoms simultaneously. Table 2 provides suggestions on how frequently surveillance should be conducted on patients with HHT.

**Table 2:** Multisystem surveillance in HHT. This table outlines general surveillance strategies for organ systems commonly affected in HHT, including recommended screening modalities and intervals based on the Second International HHT guidelines [6]. These recommendations represent standard care practices and are not specific to the presented patient. Abbreviations: AVMs = arteriovenous malformations; CT = computed tomography; MRI = magnetic resonance imaging; GI = gastrointestinal; EGD = esophagogastroduodenoscopy; HHT = hereditary hemorrhagic telangiectasia. Source: Table created by MS and JRP with data extracted from [10].

Organ system	Common manifestation	Screening modality	Recommended interval
Pulmonary	AVMs	Contrast echocardiography or CT chest	At diagnosis, repeat every 5 years per guidelines
Cerebral	AVMs	MRI brain	One time at diagnosis
Hepatic	AVMs	Doppler ultrasound/CT	Symptom based
GI	Telangiectasia	EGD/colonoscopy ± capsule endoscopy	If anemia or bleeding

**Figure 4:**
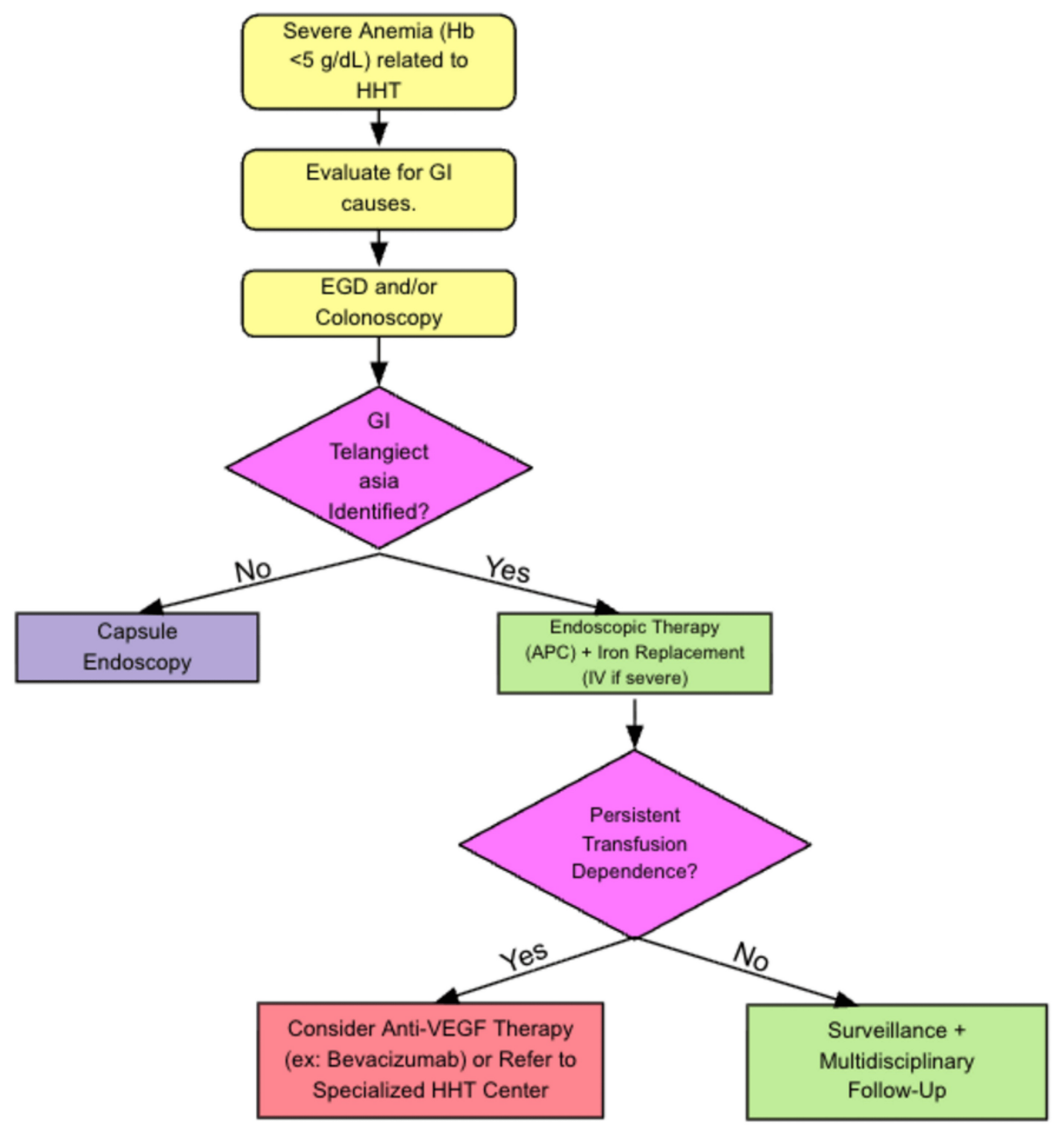
Management algorithm for refractory HHT-related anemia. This flowchart presents a simplified, guideline-based approach to the management of severe or refractory anemia in patients with HHT. It outlines general treatment strategies, including iron replacement, endoscopic therapy, and consideration of systemic therapies. This algorithm reflects published management approaches and is not specific to the presented case. Abbreviations: Hb = hemoglobin; HHT = hereditary hemorrhagic telangiectasia; TSAT = transferrin saturation; MCV = mean corpuscular volume; GI = gastrointestinal; EGD = esophagogastroduodenoscopy; APC = argon plasma coagulation; IV = intravenous; Anti-VEGF = anti-vascular endothelial growth factor. Source: Flowchart created by MS and JRP with data extracted from [3].

Future research should focus on treatments that last longer and prevent recurrences, or newer gene-targeted therapies that may help fix the underlying issues, making the solution more permanent. Advances in these areas may significantly reduce recurrence rates and transfusion dependence in patients with severe HHT. Figure 4 demonstrates a way to manage refractory HHT-related anemia.

## Conclusions

This case highlights the immense consequences that arise from HHT-related GI bleeding and the importance of early identification and treatment of visceral AVMs. Patients with HHT require lifelong monitoring of anemia, GI bleeding, and cardiopulmonary complications. Endoscopic ablations, iron therapy, and transfusion support are critical to prevent high-output heart failure. The need for persistent screening and individualized treatment protocols for HHT is even more prevalent given the chronic and progressive nature of the disease and its associated morbidity. Transfusion-dependent, multisystem HHT serves as a warning to clinicians that severe anemia may reflect progressive systemic vascular disease that requires early intervention and consideration of advanced therapies.
